# Breast cancer expression of YKL-40 correlates with tumour grade, poor differentiation, and other cancer markers

**DOI:** 10.1038/bjc.2011.347

**Published:** 2011-09-20

**Authors:** R Shao, Q J Cao, R B Arenas, C Bigelow, B Bentley, W Yan

**Affiliations:** 1 Pioneer Valley Life Sciences Institute, 3601 Main Street, Springfield, MA 01199, USA; 2 Molecular and Cellular Biology Program, Morrill Science Center, University of Massachusetts, Amherst, MA 01003, USA; 3 Department of Veterinary and Animal Sciences, University of Massachusetts, Amherst, MA 01003, USA; 4 Department of Pathology, Baystate Medical Center, Tufts University, Springfield, MA 01199, USA; 5 Department of Surgery, Baystate Medical Center, Tufts University, Springfield, MA 01199, USA; 6 Division of Biostatistics and Epidemiology, Department of Public Health, University of Massachusetts, Amherst, MA 01003, USA

**Keywords:** YKL-40, breast cancer biomarker, clinical outcome, invasive ductal carcinoma, breast ductal carcinoma *in situ*

## Abstract

**Background:**

Serum levels of a secreted glycoprotein YKL-40 are elevated in patients with a wide range of cancers including breast, colorectal, and ovarian cancers. Furthermore, these increased levels correlate with poorer survival of cancer patients, suggesting that serum levels of YKL-40 might be a prognostic biomarker. However, the tissue expression of YKL-40 and its relationship with clinical outcomes and other potential markers are poorly understood.

**Methods:**

Tissue samples from invasive breast cancers, breast ductal carcinoma *in situ* (DCIS), and cancer-free reduction mammoplasty were enrolled. YKL-40 expression was measured using immunohistochemistry and evaluated by a semi-quantification assay. Statistical analyses explored the relationship of YKL-40 with clinical outcome and other breast cancer biomarkers.

**Results:**

Breast ductal carcinoma *in situ* expressed low and moderate levels of YKL-40. In the subset of 203 patients with invasive cancer, YKL-40 levels were positively correlated with tumour grade (*P*<0.0001) and Her2/*neu* (*P*<0.01), but negatively correlated with oestrogen (*P*<0.0001) and progesterone receptor (*P*<0.0001). YKL-40 levels were inversely correlated with expressions of GATA3 (*P*=0.0137) and E-cadherin (*P*=0.0417).

**Conclusion:**

These data demonstrate that expression levels of YKL-40 are associated with tumour grade, poor differentiation, and other breast cancer markers, highlighting that tissue levels of YKL-40 serve as a valuable biomarker for breast cancer diagnosis and prognosis.

Breast cancer is the most incident cancer among women in the United States. Over the past three decades, we have come a long way towards understanding better how to prevent and treat breast cancer. As a result, fewer women now die from this disease (Jatoi and Miller, 2003; Blackman and Masi, 2006). However, we still have about 180 000 new cases diagnosed with breast cancer each year. While early treatment by surgical resection, chemotherapy, and radiotherapy can effectively prevent cancer progression and help >95% of patients survive longer than 5 years, this disease continues to be devastating for its suffers. Thus, identifying key molecules that may serve as reliable biomarkers for the diagnosis and as therapeutic targets is of paramount importance.

YKL-40 (also named human cartilage glycoprotein-39 or chitinase-3-like-1) is a phylogenetically conserved heparin- and chitin-binding glycoprotein and is classified in the family of mammalian chitinase-like proteins (Renkema *et al*, 1998; Fusetti *et al*, 2003). However, YKL-40 lacks chitinase/hydrolase activity due to mutation of an essential glutamic acid to leucine in the chitinase-3-like catalytic domain (Renkema *et al*, 1998; Fusetti *et al*, 2003). YKL-40 is expressed in several mammal types including human (Hakala *et al*, 1993), porcine (Shackelton *et al*, 1995), cow (Rejman and Hurley, 1988), mouse (Lian *et al*, 2006), rabbit (De Ceuninck *et al*, 2001), and goat (Mohanty *et al*, 2003). In humans, the expression of YKL-40 is restricted to a few cell types including chondrocytes (Hu *et al*, 1996), synoviocytes (Nyirkos and Golds, 1990), vascular smooth muscle cells (Shackelton *et al*, 1995), macrophages (Rehli *et al*, 1997), and neutrophils (Kzhyshkowska *et al*, 2007). The biophysiological activity of YKL-40 is poorly understood, however, even though its crystal structure has been illustrated (Fusetti *et al*, 2003). It is currently known that YKL-40 mediates proliferation of chondrocytes and fibroblasts (De Ceuninck *et al*, 2001; Recklies *et al*, 2002). It is also capable of promoting macrophage differentiation (Rehli *et al*, 1997) and regulating extracellular matrix remodelling (Recklies *et al*, 2002; Badariotti *et al*, 2006; Bigg *et al*, 2006), suggesting that YKL-40 functions as a tissue remodelling factor.

Several independent studies have shown that YKL-40 in serum is elevated in a wide range of human carcinomas, including breast (Jensen *et al*, 2003), colorectum (Cintin *et al*, 1999), ovary (Hogdall *et al*, 2003), prostate (Kucur *et al*, 2008), brain (Pelloski *et al*, 2005), and blood (Bergmann *et al*, 2005). These increased levels have also been observed to be associated with poorer survival of cancer patients (Cintin *et al*, 1999, 2002; Hogdall *et al*, 2003; Jensen *et al*, 2003; Johansen *et al*, 2003; Bergmann *et al*, 2005; Pelloski *et al*, 2005), suggesting that serum levels of YKL-40 are a prognostic cancer biomarker. In breast cancer, increased serum levels of YKL-40 were found in 19% of patients with primary cancer (Johansen *et al*, 2003) and 30% of patients with metastatic cancer (Jensen *et al*, 2003), supporting the notion that YKL-40 is associated with cancer aggressiveness. We have recently identified that the expression levels of YKL-40 are associated with tumour vascular formation in breast cancer and in brain tumours, demonstrating its angiogenic properties in cancer development (Shao *et al*, 2009; Francescone *et al*, 2011). It remains to be determined whether YKL-40 is an independent biomarker or associated with other potential breast cancer biomarkers, and whether expression of YKL-40 is closely associated with clinical outcomes.

In this study of 203 cases of invasive cancer, we took advantage of a recently established polyclonal antibody specifically recognising YKL-40 to evaluate the expression of YKL-40 in different breast tissues including benign breast tissue, breast ductal carcinoma *in situ* (DCIS), and invasive ductal carcinoma (IDC) using an immunohistochemical (IHC) assay. We then employed a semi-quantitative approach to quantify the expression levels of YKL-40 and analysed the relationship between expression levels of YKL-40 and clinical outcomes. In addition, we investigated the association of YKL-40 with other two potential biomarkers GATA-binding protein 3 (GATA3) and E-cadherin in breast cancer, both of which correlate with ductal differentiation and a favourable prognosis of breast cancer (Bertucci *et al*, 2000; Graff *et al*, 2000; Garcia-Closas *et al*, 2007; Kouros-Mehr *et al*, 2008a). GATA3 is a zinc-finger transcription factor that has been shown to drive E-cadherin expression (Ko and Engel, 1993; Yan *et al*, 2010). E-cadherin is expressed in the cell membrane; it regulates cell–cell tight contacts and inhibits tumour cell motility as a tumour suppressor (Cheng *et al*, 2001; Wong and Gumbiner, 2003).

## Materials and methods

### Collection of cancer samples and clinical data

We retrospectively collected tissue samples from 221 breast cancer patients that underwent surgical therapy at Baystate Medical Center between 1994 and 2008. This random selection was primarily dependent on the availability of follow-up medical record over 10 years. Of these, 203 patients were diagnosed with invasive cancer and 18 patients were diagnosed with DCIS. Cancer samples (59 IDCs) available between year 2001 and 2002 were selected for the IHC analysis of GATA3 and E-cadherin protein expressions. Three normal tissue samples of reduction mammoplasty available at the Department of Pathology were also selected for the study as normal controls. All tissue specimens were maintained at the Department of Pathology and patient medical records were available at Baystate Cancer Center. The medical chart review abstracted information on ages, diagnosis, and pathological data such as tumour stages and grade in addition to status of lymph nodes and distant organ or tissue metastases. Ectopic cancer metastases were mainly diagnosed from CT scan, MRI, or PET-CT, demonstrating existence of tumours that were not anatomically connected to the primary breast cancer such as lung, liver, bone, chest wall, brain or bowel carcinoma. Most of these metastases were diagnosed at the later period of the disease. This study was approved by both the Baystate Medical Center and University of Massachusetts Amherst Institutional Review Board.

### IHC staining

Paraffin-embedded human cancer specimens were cut to 6 *μ*m thickness and processed for the staining of YKL-40, GATA3, E-cadherin, ER, PR, and Her2/*neu*. In brief, the samples were incubated with 3% H_2_O_2_ to block endogenous peroxidase activity for 30 min followed by incubation with blocking buffer containing 10% goat serum for 1 h. Then, one of antibodies such as rabbit anti-YKL-40 (1 : 400), ER (clone 6F11), PR (clone 1A6), or Her2/*neu* (clone CB11) (pre-diluted antibodies, Ventana Inc., Tucson, AZ, USA), mouse anti-GATA3 (1 : 200, Santa Cruz Inc., Santa Cruz, CA, USA), or anti-E-cadherin (1 : 2000, Invitrogen, Carlsbad, CA, USA) antibodies was incubated at room temperature for 2 h. A goat anti-rabbit or anti-mouse secondary antibody (1 : 100) conjugated with HRP was added. Finally, DAB substrate (Dako Inc., Carpinteria, CA, USA) was introduced for several minutes and after washing, methyl green will be used for counterstaining.

### YKL-40, GATA3, and E-cadherin scoring

YKL-40 staining was evaluated as sum of two scores based on percent and intensity of positive staining cells as following (1) percent: no staining is 0 points, <10% of cells stained is 1 point, 11–50% of cells stained is 2 points, and >50% of cells stained is 3 points; (2) intensity: no staining is 0 points, weak staining is 1 point, moderate staining is 2 points, and strong staining is 3 points. Thus, the valid range of scores was 0–6 from combined density and intensity analyses. For statistical analysis, YKL-40 scores were further classified into three groups: negative/low (0–2 points), medium (3–4 points), and high (5–6 points) of YKL-40 staining. Finally, in analyses of YKL-40 and its relationship with GATA3 and E-cadherin, 0–2 points and 3–6 points of YKL-40 were designated as negative and positive, respectively. GATA3 and E-cadherin were evaluated using staining intensity as low/weak or high/strong.

### Statistical analysis

Fisher's exact was used to assess the statistical significance of apparent co-variations of YKL-40 and each pathological factor and biomarker. We report two-sided significance levels. All analyses were performed using Stata version 10.1 (StataCorp, College Station, TX, USA). A Kaplan–Meier curve was tested for patient survival followed by a log-rank test analysis.

## Results

### Specificity of an anti-YKL-40 antibody and expression of YKL-40 in benign breast tissue and DCIS

**Figure 1:**
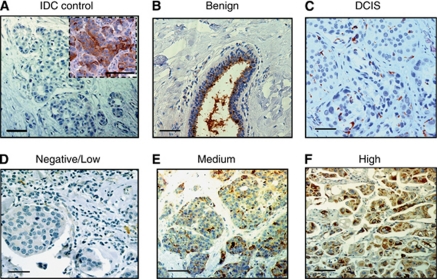
YKL-40 expression in breast cancer. A negative control of IDCs was shown once rAY was pre-incubated with recombinant YKL-40 at a molar ratio of 1 : 1 before application in a cancer specimen (**A**). Insert indicated the positive staining without pre-incubation with recombinant YKL-40. In benign mammary tissue, YKL-40 secreted from ductal epithelial cells was accumulated in the apical aspect of duct lumen (**B**). A representative case of DCIS indicated a low level of staining of YKL-40 (**C**). Representative examples of immunohistochemical staining for negative, medium, and high expression levels of YKL-40 were shown in (**D**), (**E**), and (**F**), respectively. Bars: 100 *μ*m.

To analyse expression levels of YKL-40 in cancers, we began testing the specificity of an affinity-purified, polyclonal anti-YKL-40 antibody (rAY) in IHC assay. The rAY was pre-incubated with recombinant YKL-40 overnight before applying to breast cancer samples that demonstrated strong expression of YKL-40 in a pretest (Figure 1A, insert). This pre-incubation with YKL-40 recombinant protein fully prevented the binding of rAY with YKL-40 present in the cancer tissue as a result of no YKL-40-specific signal detected (Figure 1A). Pre-immune serum rabbit IgG as a negative control did not recognise any signals in the cancer tissue either (data not shown). We then tested YKL-40 expression in a few cases of both benign mammary tissue and breast cancer tissue at early stages. Benign mammary tissue expressed varied levels of YKL-40 as one of three subjects did not express YKL-40 (data not shown); whereas the other two cases expressed YKL-40 that was considerably accumulated in the apical surface of the epithelial ducts (Figure 1B), suggesting the secreted property of YKL-40 from ductal epithelial cells. In DCIS, YKL-40 immunoreactivity was mainly found in the cytoplasm of cancer cells (Figure 1C). These data indicate that rAY can serve as a powerful tool to evaluate expression of YKL-40 in breast tissue.

In a pretest, we found that YKL-40 was not ubiquitously expressed by cancers in the same density or intensity; instead, its expression pattern commonly displayed diverse levels in each tissue specimen (data not shown). In order to precisely evaluate expression levels of YKL-40 in cancer tissue, a semi-quantification system assaying both density and intensity of the staining was engaged. Quantitative analysis of YKL-40 in 18 cases of DCIS demonstrated that 7 and 11 cases expressed low and medium levels of YKL-40, respectively; but none of cancers exhibited strong expression of YKL-40 (Figure 1C). These data indicate that breast cancer at early stages express low or medium levels of YKL-40.

### Characteristics of invasive cancer samples

Study participants were between 23 and 88 years of age with a median of 56.5 years (Table 1). The majority of the cancers were ductal carcinomas (81.3%, IDC) followed by lobular carcinomas (12.3%) and medullary carcinomas (0.5%). The remaining 12 cancers (5.9%) involved infiltrating pleomorphic lobular carcinoma and inflammatory cancer (Table 1). Most were diagnosed between stage I (45.3%) and stage II (43.8%). The median tumour size was ∼2–5 cm (50.2%). Local (e.g., lymphatic nodes) and distant metastases (e.g., lung, liver, or brain) were found in 35.2% and 20.2% of these patients, respectively.

Figure 1D–F was representative examples of three different expression levels of YKL-40. In all, 121 of 203 cases (59.6%) exhibited negative or low expression of YKL-40; 82 patients (40.4%) were YKL-40 positive (Table 1). Forty-three patients (21.1%) displayed strong expression of YKL-40 and thirty-nine patients (19.2%) expressed medium levels of YKL-40. Older age was associated with prevalence of invasive cancers, regardless of YKL-40 levels.

### Associations of YKL-40 with clinical–pathological factors

**Figure 2:**
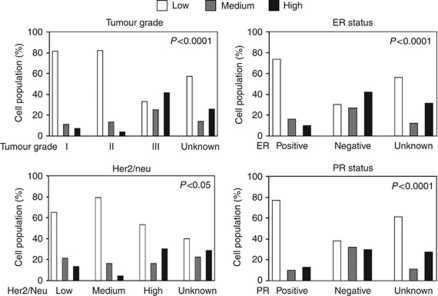
Relationship between YKL-40, tumour grade, and expression levels of ER, PR, or Her2/neu. The data of different expression levels of YKL-40 in tumour grade, ER, PR and Her2/*neu* listed in Table 2 were analysed graphically. Fisher's exact was used to test the significance and *P*-values were provided.

**Figure 3:**
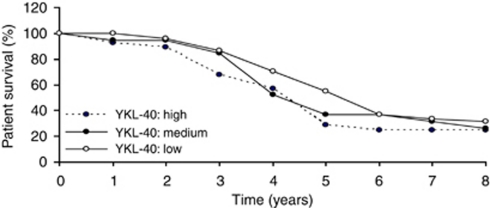
YKL-40 expression is not associated with patient overall survival. Patient survival data available in 8-year follow-up medical record were analysed using a Kaplan–Meier survival curve. A log-rank test did not show a significant difference in groups containing high (*n*=28), medium (*n*=19), or low (*n*=51) levels of YKL-40.

We observed no statistically significant association of YKL-40 with any of age, tumour stage, tumour size, node status, or distant metastasis (Table 1). Histological subtype was marginally statistically associated with YKL-40 (*P*-value=0.058); this likely reflects the higher prevalence of high YKL-40 expression in the IDC group. YKL-40 expression was strongly associated with tumour grade (Table 1; Figure 2; *P*<0.0001), reflecting the higher prevalence of high YKL-40 scores in the tumour grade III group, but with no evidence of trend. YKL-40 was also associated with expression of Her2/*neu* (*P*<0.01; Figure 2); this similarly reflects the higher prevalence of high YKL-40 scores in Her2/*neu* subgroup, but without evidence of trend. In contrast, the levels of YKL-40 were inversely correlated with both ER and PR levels (Table 1; Figure 2; *P*<0.0001). However, there was no significant difference between YKL-40 expression and patient overall survival (Figure 3) or disease-free survival (data not shown) in 8-year follow-up studies. Collectively, these data demonstrate that expression levels of YKL-40 were positively correlated with tumour grade and Her2/*neu*, but negatively correlated with ER and PR expressions.

### Associations of YKL-40 with GATA3 and E-cadherin

**Figure 4:**
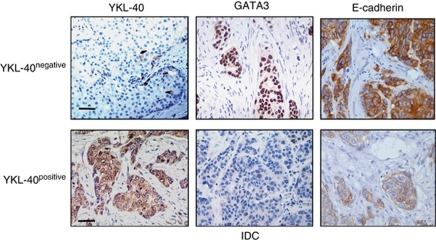
An inverse correlation of YKL-40 with GATA3 and E-cadherin. Specimens from 59 IDC patients between year 2000 and 2002 were processed for immunohistochemistry of YKL-40, GATA3, and E-cadherin. Representatives of negative and positive of YKL-40 with corresponding high and low staining of GATA3 and E-cadherin were shown. GATA3 was stained in the nucleus and E-cadherin was located on cell membrane. Bar: 100 *μ*m.

We observed negative associations of YKL-40 with both GATA3 and E-cadherin (Figure 4; Table 2). Samples with low or negative levels of YKL-40 exhibited high expressions of both GATA3 and E-cadherin; whereas cancers with high or positive levels of YKL-40 indicated low or negative levels of GATA3 and E-cadherin, *P*=0.0137 and *P*=0.0417, respectively (Table 2). In addition, an analysis of ER with GATA3 and E-cadherin showed a strong correlation between ER and GATA3 or E-cadherin (Table 2). These data support the notion that expression levels of YKL-40 are inversely associated with cancer differentiation.

## Discussion

Accumulating evidence from clinical studies of breast cancers has demonstrated that elevated serum levels of YKL-40 correlate with cancer progression and decreased disease-free survival (Johansen *et al*, 1995; Jensen *et al*, 2003). Thus, serum levels of YKL-40 are suggested as a prognostic cancer biomarker. However, little is known concerning the tissue source for YKL-40 production, which may be the determinant of serum levels of YKL-40 in cancer. We currently found that approximately a half (21.1%) of YKL-40-positive cancers (40.4%) expressed strong YKL-40, the population of which may account for the evidence reported previously that 20–24% of patients demonstrate elevated serum levels of YKL-40 in overall breast cancer population (Johansen *et al*, 1995, 2003). Interestingly, the tissue expression levels of YKL-40 were not directly correlated with patient survival, suggesting that cancer cells may not serve as the only source responsible for serum concentrations of YKL-40. Indeed, YKL-40 is also produced by other cells such as vascular smooth muscle cells (Shackelton *et al*, 1995), macrophages (Rehli *et al*, 1997), and neutrophils (Kzhyshkowska *et al*, 2007). Strong expression of YKL-40 by vascular cells, mast cells, neutrophils, and macrophages associated with cancer cells was found in breast cancer samples (Roslind *et al*, 2007 and our unpublished data). Thus, all these cells may together contribute to the blood levels of YKL-40, although we currently cannot rule out other sources besides tumour tissue. Nevertheless, we identified the tissue level of YKL-40 is correlated positively with another breast cancer marker Her2/*neu* and negatively with other markers ER, PR, GATA3, and E-cadherin, highlighting the valuable biomarker of YKL-40 in breast cancer diagnosis.

Here, we used a semi-quantified analysis assaying both staining density and intensity, which can precisely evaluate varied expression levels of YKL-40 in each specimen. Some of the data were consistent with a previous report that showed correlation of increased expression levels of YKL-40 with tumour grade, poor differentiation, and decreased disease-free survival (Kim *et al*, 2007). Interestingly, another clinical study gave rise to opposite evidence that elevated expression of YKL-40 was associated with positive levels of ER and PR, but did not correlate with patient survival (Roslind *et al*, 2007). This discrepancy may be attributed to the different quantification analyses by which that study assessed only the expression intensity of YKL-40 in cancers. In addition, different agents engaged for IHC analysis may be another contributor to the inconsistency. For example, different affinity-purified anti-YKL-40 antibodies may underestimate the expression of YKL-40. In the current study, we used multiple staining approaches to validate rAY as an appropriate tool that can specifically recognise YKL-40 in the tissue, before we examined YKL-40 expression in cancer specimens. We substantially analysed this antibody specificity using different positive and negative controls, and pre-incubation of tissue with recombinant YKL-40 that sufficiently prevents the binding of rAY with YKL-40 in cancer. In addition, we also tested the interaction of rAY with recombinant protein YKL-40 or YKL-40 secreted from tumour cells in an immunoblotting assay (e.g., an osteoblastoma line MG-63 and a brain tumour line U87) and we found that its specificity was identical to that observed in IHC (data not shown), confirming the high specificity of rAY.

The traditional evaluation of breast cancer prognosis is mainly dependent on primary tumour size and the status of invaded axillary lymph nodes. Other measurements such as the status of ER and PR, and the degree of tumour differentiation also indicate some value in assisting cancer diagnosis and prognosis. Over the past decades, accumulating studies have demonstrated that elevated expression of transmembrane protein kinase Her2/*neu* is associated with poorer prognosis (Willsher *et al*, 1996; Jensen *et al*, 2003). Testing Her2/*neu* as the prognostic utility has been directed to devise anti-Her2/*neu* therapies (Chang, 2010; Junttila *et al*, 2010). Interestingly, there is strong evidence showing that serum levels of YKL-40 and Her2/*neu* independently reflect cancer metastasis (Jensen *et al*, 2003). It is noteworthy that YKL-40 serum levels indicated a stronger predictor of patient survival than Her2/*neu* or others (e.g., negative ER). This may be attributed to the angiogenic signature of YKL-40 in tumour metastasis, which fuels tumour cell proliferation and invasiveness through facilitating blood vessel formation (Shao *et al*, 2009). Thus, YKL-40 could not only serve as a prognostic marker, but also as a target for cancer therapy. Apart from these biomarkers for breast cancer, mounting evidence also indicated that expressions of GATA3 and E-cadherin in breast cancer are strongly associated with ductal differentiation and a favourable prognosis, suggestive of new breast cancer biomarkers (Mehra *et al*, 2005; Eeckhoute *et al*, 2007; Voduc *et al*, 2008; Kouros-Mehr *et al*, 2008b). In the context with these data, here we have identified that YKL-40 expression is negatively associated with GATA3, E-cadherin, ER, and PR; therefore, we demonstrate that YKL-40 together with other markers including Her2/*neu*, ER, PR, GATA3, and E-cadherin could serve as a powerful tool for the diagnosis and prognosis of breast cancer as well as a potential target for future cancer therapy.

## Figures and Tables

**Table 1:** Relationship between YKL-40 scores and clinical–pathological factors

		**No. of YKL-40 scores^a^ (%)**			
**Factor**	**No. (%)**	**Low**	**Medium**	**High**	***P***
*Age (years)*					0.647
<40	13 (6.4)	7 (53.8)	1 (7.7)	5 (38.5)	
40–49	43 (21.2)	27 (62.8)	10 (23.5)	6 (13.9)	
50–59	55 (27.1)	33 (60)	11 (20)	11 (20)	
>60	92 (45.3)	54 (58.7)	17 (18.5)	21 (22.8)	
Total	203 (100)	121 (59.6)	39 (19.2)	43 (21.2)	
					
*Histological subtype*					0.058
Ductal carcinoma	165 (81.3)	90 (54.5)	34 (20.6)	41 (24.8)	
Lobular carcinoma	25 (12.3)	20 (80)	4 (16)	1 (4)	
Medullar carcinoma	1 (0.5)	1 (100)	0	0	
Other	12 (5.9)	10 (83.3)	1 (8.3)	1 (8.3)	
					
*Tumour stage*					0.554
I	92 (45.3)	58 (63)	19 (20.6)	15 (16.3)	
II	89 (43.8)	51 (57.3)	16 (17.9)	22 (24.7)	
III	19 (9.4)	9 (47.3)	4 (21)	6 (31.6)	
Unknown	3 (1.4)	3 (100)	0	0	
					
*Tumour size (cm)*					0.407
<2	84 (41.4)	55 (65.5)	15 (17.9)	14 (16.7)	
2–5	102 (50.2)	58 (56.9)	21 (20.6)	23 (22.5)	
>5	15 (7.4)	6 (40)	3 (20)	6 (40)	
Unknown	2 (1)	2 (1.6)	0	0	
					
*Tumour grade*					<0.0001*
I	27 (13.3)	22 (81.5)	3 (11.1)	2 (7.4)	
II	74 (36.5)	61 (82.4)	10 (13.5)	3 (4)	
III	95 (46.8)	34 (35.8)	25 (26.3)	36 (37.9)	
Unknown	7 (3.4)	4 (57.1)	1 (14.3)	2 (25.6)	
					
*Her2/neu*					0.010^*^
Low	37 (18.2)	24 (64.9)	8 (21.6)	5 (13.5)	
Medium	43 (21.2)	34 (79)	7 (16.3)	2 (4.6)	
High	43 (21.2)	23 (53.5)	7 (16.3)	13 (30.2)	
Unknown	80 (39.4)	40 (50)	17 (22.2)	23 (28.7)	
					
*ER status*					<0.0001^*^
Positive	128 (63)	94 (73.4)	21 (16.4)	13 (10.1)	
Negative	59 (29)	18 (30.5)	16 (27.1)	25 (42.4)	
Unknown	16 (7.9)	9 (56)	2 (12.5)	5 (31.2)	
					
*PR status*					<0.0001^*^
Positive	101 (49.7)	78 (77.2)	10 (9.9)	13 (12.9)	
Negative	84 (41.4)	32 (38.1)	27 (32.1)	25 (29.8)	
Unknown	18 (8.9)	11(61.1)	2 (11.1)	5 (27.7)	
					
*Node status*					0.122
Positive	81 (35.2)	48 (59.2)	15 (18.5)	18 (22.2)	
Negative	111 (54.7)	64 (57.6)	22 (19.8)	25 (22.5)	
Unknown	11 (5.4)	11 (100)	0	0	
					
*Distant metastasis* b					0.613
Positive	41 (20.2)	24 (58.5)	11 (26.8)	6 (14.6)	
Negative	93 (45.8)	52 (55.9)	16 (17.2)	22 (23.6)	
Unknown	69 (34)	42 (60.9)	12 (17.4)	15 (21.7)	

Abbreviations: ER=oestrogen receptor; PR=progesterone receptor.

a YKL-40 low, medium, and high scores from combined intensity and density analysis are 0–2, 3–4, and 5–6 points, respectively.

b The status of metastasis was derived from diagnostic imaging tests as described in Materials and methods, in which negative stands for no metastasis identified in distant organs and unknown indicates no these tests were performed. ^*^P<0.05.

**Table 2:** Correlation of YKL-40 and ER with GATA3 and E-cadherin in IDC

	**YKL-40^a^**			**ER^b^**		
	**Negative**	**Positive**	***P*-value**	**Negative**	**Positive**	***P*-value**
*GATA3*						
Low	15	19		19	14	
High	19	6	0.0137	1	16	0.00030
						
*E-cadherin*						
Low	9	13		15	7	
High	25	12	0.0417	5	23	0.00038

Abbreviations: ER=oestrogen receptor; IDC=invasive ductal carcinoma.

a *n*=59.

b *n*=50.
